# Opinion Paper: Rationale for Supra-National Training in Neonatology

**DOI:** 10.3389/fped.2022.899160

**Published:** 2022-07-01

**Authors:** Sven Wellmann, Manfred Künzel, Pascal Fentsch, Jean-Claude Fauchère, Heike Rabe, Tomasz Szczapa, Gabriel Dimitriou, Maximo Vento, Charles C. Roehr

**Affiliations:** ^1^Department of Neonatology, University Children's Hospital Regensburg (KUNO), Hospital St. Hedwig of the Order of St. John, University of Regensburg, Regensburg, Germany; ^2^Machine Learning for Education Laboratory, Ecole Polytechnique Fédérale de Lausanne (EPFL), Lausanne, Switzerland; ^3^European Society for Paediatric Research, Satigny, Switzerland; ^4^Department of Neonatology, University Hospital Zurich, Zurich, Switzerland; ^5^Brighton and Sussex Medical School, University of Sussex, Brighton, United Kingdom; ^6^Neonatal Biophysical Monitoring and Cardiopulmonary Therapies Research Unit, Department of Newborns' Infectious Diseases, Chair of Neonatology, Poznań University of Medical Sciences, Poznań, Poland; ^7^Department of Paediatrics, University of Patras Medical School, Patras, Greece; ^8^Division of Neonatology, University and Polytechnic Hospital La Fe, Valencia, Spain; ^9^National Perinatal Epidemiology Unit Clinical Trials Unit, Nuffield Department of Population Health, Medical Sciences Division, University of Oxford, Oxford, United Kingdom; ^10^University of Bristol, Faculty of Health Sciences, Bristol, United Kingdom; ^11^Neonatal Unit, Southmead Hospital, North Bristol Trust, Bristol, United Kingdom

**Keywords:** education, master degree, physician, neonate, baby, child

## Introduction

Clinical training in neonatology takes place where neonates are cared for, at the cot-side, in neonatal units. Neonatal units vary widely in size, specialization, resources, staffing and academic level. Among them, small units make up a large proportion which have more difficulties to offer structured training courses on site, local expertise on all relevant neonatal topics, and appropriate exposition of the trainee to high-risk cases. Although evidence-based medicine is widely accepted, training of physicians, including neonatologists, often follows ineffective learning methods, or even less favorable, learning by doing. When looking at the national training requirements and standards within Europe, there are large differences between countries. Some countries have training requirements, standards and national training courses in place other countries have none of this. Therefore, it is worthwhile to create a supra-regional or even supra-national training program that complements local clinical work on an individual basis to provide structured training and evidence-based education anywhere and anytime.

The European Society for Paediatric Research (ESPR) has long been committed to the education and training of medical doctors specializing in neonatology. Together with the European Board of Neonatology (EBN), which is a substructure of ESPR devoted to the design and implementation of a syllabus that comprises the theoretical and practical needs for the European Training in Neonatology, the European Training Requirements (ETR) in Neonatology has been developed.

The 2021 updated syllabus, the current ETR in Neonatology, is based on the previous 2007 syllabus version and has been approved by the Union of European Medicine Specialist (EAMS) in April 2021. Interestingly, the 2021 syllabus content was updated by the EBN members, but also critically incorporated and comments suggestions of national representatives of 30 European countries following two sequential surveys and face to face meetings. Each country pertaining to the EBN has a different national training curriculum to achieve the training standards required to exert as neonatologists. The aim of the ETR in Neonatology has been to harmonize training requirements within Europe to achieving a basic and reliable standard of quality in theoretical knowledge and practical skills alongside the European countries.

We present an online training concept that meets the needs of neonatal training situations and implements the latest effective didactic elements.

## Analysis of the Training and Work Situation

The process of revising the 2007 syllabus and preparing the 2021 syllabus, the current ETR in Neonatology, included several expert meetings, surveys and targeted interviews to address and analyze the work and training situations of trainees in neonatology in Europe. This was done in a participatory process with the heads of neonatal and pediatric intensive care units, consultants of different levels, various medical professions such as surgeons and radiologists involved in the care of children, and educational specialists. The process was designed as action research: each participant was challenged to make a specific contribution to the new training, thereby changing and adding to the existing training practices. The premises and the outcomes were reflected upon and divided into seven categories, each offering specific insight.

1. Professional situation: Physicians specializing in neonatology do not start from scratch. They are trained physicians in pediatrics. This experience allows the use of elements of transfer didactics. It is not necessary to build up knowledge elements first to gain experience, but students can work and learn with existing routines.
2. Level of training: According to the Novice-to-Expert scheme (1, 2) (novice, advanced beginner, competent, proficient, expert) physicians specializing in neonatology are competent to proficient in many aspect. However, trainees, depending on their background, may be novice in different areas more specific to neonatology. This means for training to focus on skills acquisition only in selected areas. The training could immediately include reflective practice in general.
3. Knowledge structures: Looking at the required knowledge structures (3, 4) and use memory models, two types are needed: on the one hand, procedural knowledge, which enables diagnostic and therapeutic decisions tailored to a specific action situation; and on the other hand, declarative knowledge, which represents the knowledge space, i.e., the possible diagnostic and therapeutic measures, their possible applications, differences, advantages and disadvantages. etc.
4. Collaboration: A central element of clinical practice is inter-professionality. Although neonatologists are the case-leading physicians for neonates, different professions and specialties contribute to clinical success. Neonatologists must integrate their own activities with the routines and competences of others, and thereby make their own contribution to the management of the clinical situation. This means that cooperation, participatory management and communication are important premises of medical training in neonatology. In case-based learning sequences, not only the handling of the highly vulnerable patients is practiced, but also the collaborative management of the overall situation.
5. Multidisciplinary: Since children and in particular neonates are not legally responsible and cannot express their needs, wishes and hopes, a multidisciplinary and usual transgenerational situation arises. These situations are of mutual learning (5, 6).
6. Integration of Training with Organizational Learning: Trainees and the training itself should contribute to quality assurance and quality improvement in daily clinical work across the whole neonatal unit. Training is part of a learning organization and vice versa. Hence, training will accompany clinical practice and, conversely, the operational organization in the neonatal unit will be accompanied by training elements in which the physicians participate. Closely linked, trainee-driven projects will emerge between neonatal units and the training provider from guidelines to trainings to continuous improvement.
7. Uniqueness of Neonatal Medicine: One aspect makes training in neonatology particularly interesting and unique. It is the transition of the infant from intra to extra-uterine life. Due to the constant changes in the underlying physiological and pathological state, clinical decisions must involve few, insufficient assumptions and constant observation. This requires a great deal of experience. The challenge is to create training elements that can reduce the number of experiences in these rapidly changing situations and thus achieve a faster operational capability.

## The Requirement for Effective Training

Since the 1990s, the term ‘evidence-based medicine' is a guiding principle in clinical decision-making. It means the conscientious, explicit and reasonable use of current best evidence in the care of patients (7). Research and clinical experience of experts is reflected in evidence-based guidelines and policies. Evidence-based education (EBE) (8, 9) goes in a similar direction. Educational practices should also be based on the best available scientific evidence, rather than tradition, personal judgment, or other influences. EBE can be summarized as evidence-based teaching, evidence-based learning, and school effectiveness research.

However, training and full immersion in evidence-based medicine does not mean that evidence-based principles are also followed in education. Tradition and personal judgment noticeably prevail in medical education, and EBE is largely unknown in clinical training of physicians. Thus, medical experts rely on the latest evidence for patient care, but train their young colleagues in an eminence-based fashion or leave the training to daily clinical practice.

Hattie (10) and his team performed meta-studies to examine the efficacy of various educational settings on learning performance as compared to settings without educational interventions. They observed that learning progress occurs even without a planned training intervention, which we all know as “learning by doing.” This is more than questionable in clinical settings. Hattie et al. (11) has advanced this work in recent years. Both express the effectiveness of training settings in effect sizes (d). Beywl states that an effect size below <0 would be harmful; effects up to 0.15 can be achieved through development or experience even without training (learning by doing). Values >0.40 can be achieved by dedicated training in the real world, not only in the ideal world of research settings. Problem-based learning is slightly more effective with *d* = 0.26 than PowerPoint lectures (*d* = 0.15). Unfortunately, the effect of both methods is not measurable in the real world. To achieve significant effects in the real world, training should consist of elements such as performance assessment by professionals (*d* = 1.29), microteaching (*d* = 0.88), and cognitive task analysis using cases (*d* = 1.29) or with transfer strategies (*d* = 0.86). These all have effect values significantly above 0.15. The aim is to develop education with an effect size of *d* > 0.70 while avoiding all kinds of education with lower values e.g., non-didactic supplementary materials (*d* = 0.32) and classic case studies (*d* = 0.37).

It should be noted that learning by doing can take on different forms, depending on the level of educational intervention by a supervising practitioner. By itself, learning by doing is not necessarily deficit as an educational model, but rather in its implementation. Merritt et al. (12) presents a model for clinical education in which learning by doing is part a detailed learning circle under the guidance of experts. This type of structured and conscious apprenticeship requires education of the preceptors which may be a barrier to implementation.

The even greater challenge is to get these methods accepted and embraced in training of all types of medical specialists, e.g., in neonatology. It is easy to name a list of methods, but it is almost impossible to create medical education with elements that have an effect size of *d* > 70. In fact, the authors are convinced that medical education can largely be accelerated and patient care improved, not only in neonatology, if evidence-based education becomes the standard. Thus, the novelty of this communication is that a working group for medical education representing multiple nations across Europe agree not only on the importance of EBE but also on the feasibility of implementation and further created tools for implementation. Consequently, appropriate materials for online modules have been developed.

A summary of powerful methods used in EBE are the following: Individual exercises are structured to promote transfer strategies (*d* = 0.86). We evaluate the individual participant contributions and how far they were thought over (control of learning effort *d* = 0.77). Practical experiences and procedures are shared and analyzed in reciprocal learning (*d* = 0.74) with scaffolding by tutors (*d* = 0.82). Processing consists of making summaries (*d* = 0.79), elaborating and organizing concepts. Decision trees are used in own and reported situations (*d* = 0.75). For the assignments we provide elaborated didactic supporting material (*d* = 0.72) that is adapted to different levels (to prior knowledge, *d* = 0.93). For certain complex tasks, individual group members work out different aspects in subject groups and then return to their home group (group puzzle, *d* = 1.20).

## The Overall Concept of the Supra-National Online Master's Program in Neonatology

The newly developed online program in neonatology accompanies individual clinical training and implements our research findings and latest evidence-based education knowledge in effective learning processes. The rational for the involvement of supervisors, tutors, peer groups, lecturers, other professions and the commitment of parents is summarized in the following paragraphs and in Table 1.

**Table 1:** Training techniques in the master of neonatology.

**What**	**Who**	**Where**	**How**
1) Constant performance assessment	Supervisor	Hospital, clinical workplace	Regular evaluation of implementation of tasks in clinical practice, documentation in a portfolio and plot of the progress
2) Microteaching, cognitive task analysis and transfer strategies	Tutor	Online	Illustrated and animated cases requiring step-by-step decisions by the student. If needed the student can consult basic knowledge resources provided before entering the decision. The whole process is filed and visible to the tutor, and thus can later be taken up in microteaching or assigned to peer group discussions.
3) Reflective or reciprocal learning	Peer group	Online	Student solutions and portfolio cases are analysed by peer groups in reflective or reciprocal learning
4) Decision-making related to diagnostic procedures, therapeutic or supportive interventions	Tutor, Peer group	Online	In each case scenario, only the so far completed and current information is visible to the student with a question about the next decision (e.g., what are your top 5 answers or what specific additional information you need?) and usually an inter-professional aspect is addressed (what do you expect the nurse to do?). The respective answer triggers the next step
5) Inter-professional learning	Parents, health care professionals	Online, hospital, clinical workplace	In special sessions parents will present their own experiences. Mixed professional peer groups can develop strategies for their practice and clinical setting on how to improve.
6) Targeted knowledge transfer	Invited lecturers	Online	Specific topics are presented in catchy webinars and discussions moderated by experts engage students and tutors likewise
7) Monitor with the concept of Entrustable Professional Activities (EPA)	Supervisor	Hospital, clinical workplace	EPA guide a graded increase in autonomy and responsibility toward readiness for the unsupervised practice of key tasks of the profession. In our master's programme goals are formulated as EPAs, place decision-making as the basis for entrusted activities at the center of learning, and also adopt the EPA evaluation

First, constant performance assessment (*d* = 1.29) is highly effective and, based on it, numerous training components can be individualized in a way we all know from personalized sport programs, e.g., in the gym. For the performance assessment, the trainee appoints a specific supervisor in the workplace who will act as the main preceptor for each trainee. The Supervisor will also be consulted for all aspects of the learning organization, such as the updating or creation of standards, the organization of internal trainings by the students, or the implementation of continuous improvements as a training project.

Second, online small group tutoring is used to implement strategies including microteaching (*d* = 0.88) (13), cognitive task analysis using cases (*d* = 1.29) (14, 15), transfer strategies (*d* = 0.86) (16) and scaffolding. The program works with regional groups and a tutor who knows the regional conditions and can guide the transfer of the learning concepts taking into account regional, organizational, hierarchical, technical, cultural and other conditions. Tutors are neonatologists with a strong social commitment and training experience, who can empathize with the trainees and coach them in small groups comprising no more than 5 participants. A second type of transfer strategies is important: trainees in neonatology already have clinical experience with children. They can therefore learn through transfer strategies from earlier experiences and do not need to build up basic knowledge first. Immediately after each decision, feedback is given in the form of the decision of an experienced neonatologist. This allows the trainee to compare the approach and adapt procedures in decision making to the new situation in neonatology. Only if the student cannot understand the new procedure he will consult the basic resources.

Third, peer groups are useful for reflecting on one's own solutions in given cases or one's own clinical routines in a friendly but fostering atmosphere. Participants are analyzed in reflective or reciprocal learning (*d* = 0.74) sessions (17, 18). These settings are highly effective in changing routines, attitudes and values, provide feedback and promote socialization (19, 20).

Fourth, one of the main activities of neonatologists is decision-making related to diagnostic procedures and therapeutic or supportive interventions in quickly changing situations. To address this specifically we use a procedure that we call step-by-step cases. In each case, only the current information is presented (on the phone you learn…) with a question about the first decisions (what do you ask?) and usually an inter-professional aspect (what do you expect the nurse to do?). Only after entering the answer the next step of the case is presented to the student. Depending on the case, the student receives after each step or at the end of the case story the information what colleagues did in the situation and secondly further information about the course of the case. This format is easier to implement online than on paper.

Fifth, inter-professionality is also a value-based system and needs to be addressed with training participants. This can easily happen online, when e.g., midwives and nurses participate in certain joint training modules of the physicians' continuing education. Furthermore, inter-professional learning enriches the educational outcomes of all involved persons and creates real world situations. Parents will present their own experiences. Peer group can develop strategies for their practice and clinical setting on how to improve.

Sixth, albeit power point presentations and webinars have an effect size of <0.4, we will use webinars coupled to moderated discussion to provide topical insights and latest summaries from dedicated lecturers in specific fields to a broader audience as targeted knowledge transfer.

Seventh, Entrustable Professional Activities (EPAs) were introduced to operationalize competency-based medical education and to facilitate the guidance and evaluation of learners in clinical workplaces (21). The EPA concept aims to guide a graded increase in autonomy and responsibility toward readiness for the unsupervised practice of key tasks of the profession. Entrustment decision-making has received much attention recently (22).

In our master's programme, we formulate our goals as EPAs, place decision-making as the basis for entrusted activities at the center of learning, and also adopt the EPA evaluation scale (23), which show the levels of autonomy in professional activity. We follow the tips for implementation from Peters et al. (24).

At the end of each training unit, comprising thematically structured module parts with independent learning, tutored peer group learning and other training items, there is direct online contact with specialists. During the modules, questions, uncertainties and solutions are collected—always with the projection by the tutor that these should be clearly formulated for the final meeting with the specialist. This creates commitment and deepens the discussion.

Table 1 summarizes all training techniques applied in the Master of Neonatology. They are used by tutors, peer groups or guests in online training and supervisors in the connected clinical training settings.

## Conclusion

After more than 2 years of work field analyses and development, we conclude from the feedback received by the authors, instructors and experts so far, that a supra-national online training program can meaningfully improve clinical work and that even sophisticated concepts can be translated with professional guidance into online materials, assignments and organizational structures.

The training organization with regional tutors, local supervisors and other professions involved and the tailored contributions by keynote lectures are subject to final tests. The end result will be a suitable concept for personalizing medical education adapted to the respective regional care situation despite the online setting and supranational organization.

## Author Contributions

All authors listed have made a substantial, direct, and intellectual contribution to the work and approved it for publication.

## Funding

Publication costs of this work was supported by the European Society for Paediatric Research, 1242 Satigny, Switzerland.

## Conflict of Interest

The authors declare that the research was conducted in the absence of any commercial or financial relationships that could be construed as a potential conflict of interest.

## Publisher's Note

All claims expressed in this article are solely those of the authors and do not necessarily represent those of their affiliated organizations, or those of the publisher, the editors and the reviewers. Any product that may be evaluated in this article, or claim that may be made by its manufacturer, is not guaranteed or endorsed by the publisher.
